# Peroxisomes and Innate Immunity: Antiviral Response and Beyond

**DOI:** 10.3390/ijms20153795

**Published:** 2019-08-03

**Authors:** Ana Rita Ferreira, Mariana Marques, Daniela Ribeiro

**Affiliations:** Institute of Biomedicine (iBiMED) & Department of Medical Sciences, University of Aveiro, 3810-193 Aveiro, Portugal

**Keywords:** peroxisomes, innate immunity, viruses, mitochondrial antiviral signaling protein (MAVS), signaling, microbes, bacteria, inflammation

## Abstract

Peroxisomes are ubiquitous organelles with well-defined functions in lipid and reactive oxygen species metabolism, having a significant impact on a large number of important diseases. Growing evidence points to them, in concert with mitochondria, as important players within the antiviral response. In this review we summarize and discuss the recent findings concerning the relevance of peroxisomes within innate immunity. We not only emphasize their importance as platforms for cellular antiviral signaling but also review the current information concerning their role in the control of bacterial infections. We furthermore review the recent data that pinpoints peroxisomes as regulators of inflammatory processes.

## 1. Introduction

Peroxisomes are dynamic, multifunctional, and ubiquitous organelles present in almost all eukaryotic cells [1]. They are bound by a single lipid membrane that surrounds a granular matrix, and their shape and size can alter in response to environmental stimuli [2,3,4]. Peroxisomes are crucial metabolic organelles that play important roles in lipid and reactive oxygen species (ROS) metabolism [5,6]. However, their functions are dependent on cell type, tissue, and organism. Peroxisomes interact functionally and morphologically with other organelles, such as the endoplasmic reticulum, mitochondria, or lipid droplets [7]. 

Peroxisomal dysfunctions have been linked to severe metabolic disorders in humans [4,8]. In recent years, however, it became clearer that peroxisomes also assume important nonmetabolic roles in diseases such as aging, cancer, or neurodegenerative disorders [8,9,10,11,12,13,14], as well as protective functions within the innate immune response [15,16,17].

The innate immune system is responsible for identifying threats and initiating a sequence of responses that allow the elimination of potentially infectious pathogens [18]. It involves the recognition of the pathogen by the infected cell and the production of chemical factors that lead to the recruitment of immune cells to the site of infection. It will ultimately activate the adaptive immune system and stimulate inflammation to promote healing and hamper the spread of infection [19]. 

In this review, we summarize and discuss the role of peroxisomes within innate immunity. We not only highlight their importance for the cellular antiviral response but also discuss different reports that demonstrate their role in the control of infections by other microbes. We furthermore review the role of peroxisomes as regulators of inflammatory processes.

## 2. Peroxisomes as Platforms for Cellular Antiviral Responses

**Figure 1:**
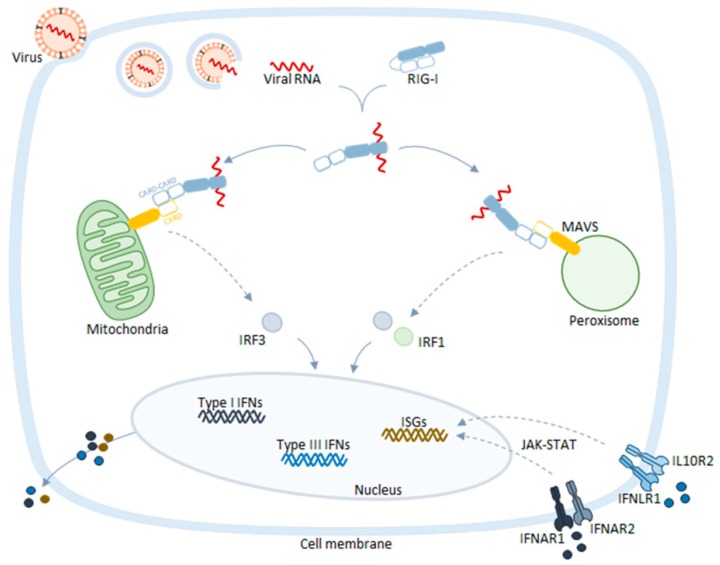
Mitochondrial antiviral signaling protein (MAVS)-dependent antiviral signaling pathway. Upon infection, viral RNA is released into the cytosol where it is sensed by retinoic-inducible gene-I (RIG-I) and/or melanoma differentiation-associated gene-5 (MDA5). These receptors travel to peroxisomes and mitochondria to activate MAVS, inducing a downstream signaling cascade that culminates with the production of type I interferons (IFNs), type III IFNs, and IFN-stimulated genes (ISGs). Once secreted, IFNs bind to specific receptors on the cell surface, activating the janus kinase/signal transducers and activators of transcription (JAK-STAT) pathway and generating an amplifying loop that results in the accumulation of different classes of ISGs. The conjugation of these responses leads to the restriction of viral replication and spreading to neighboring cells. IFNAR—interferon alfa/beta receptor complex; IFNLR—interferon lambda receptor complex; and IL10R2—interleukin-10 receptor 2.

Peroxisomes harbor the essential adaptor transmembrane protein of the retinoic-inducible gene-I (RIG-I)-like receptors (RLR) signaling, the mitochondrial antiviral signaling protein (MAVS) [20,21] (Figure 1). Upon infection, viral RNA is released into the cytosol where it is sensed by the cytosolic receptors RIG-I and/or melanoma differentiation-associated gene-5 (MDA5) [22,23,24,25]. Upon activation, these receptors travel to peroxisomes, mitochondria, or mitochondria-associated membranes (MAMs) to activate MAVS [20,21,26,27,28,29,30], through interaction via their caspase activation and recruitment domains (CARDs). This interaction induces a conformational change on MAVS, leading to the formation of resistant prion fiber-like active aggregates [31] and the subsequent amplification of downstream signaling, culminating with the production of interferons (IFNs) and IFN-stimulated genes (ISGs) that function as direct antiviral effectors [32] (Figure 1).

Dixit et al. described key differences, concerning the signaling kinetics as well as the end products, between peroxisomal and mitochondrial antiviral signaling pathways. The authors observed that peroxisomal MAVS signaling induces a rapid, but transient, type I IFN-independent expression of ISGs, while mitochondrial MAVS signaling responds with a later type I IFN-dependent and long-lasting induction of defense factors, with autocrine and paracrine effects [20]. The authors, however, discuss that these kinetic differences may be cell specific, since they were not observed in macrophages [20]. Importantly, the cooperation between peroxisomal and mitochondrial MAVS seems to be essential for a potent induction of ISGs and type I IFNs expression. When analyzing the MAVS downstream signaling, the authors further demonstrated that, although tumor necrosis factor receptor-associated factor (TRAF) 3, TRAF6, and IFN regulatory factor 3 (IRF3) were required for the signaling from both organelles, IRF1 seems to be specifically activated by peroxisomal MAVS. Further details concerning the peroxisomal signaling pathway as well as the mechanisms that drive the specific activation of IRF1 are yet to be disclosed. Dixit et al. have also found that the previously described negative regulator of MAVS, nucleotide-binding oligomerization domain-like receptor X1 (NLRX1) that is exclusively located in the mitochondria [33], does not restrict the signal from peroxisomal MAVS [20]. In a subsequent study, the same group reported that the peroxisome-dependent pathway, in addition to the induction of ISGs production, also promotes the expression of type III IFNs [34], a class of IFNs that has tissue-specific roles in antiviral immunity [35]. Moreover, they demonstrated that type III IFNs can be stimulated by a diversity of viruses, and identified peroxisomes as the signaling platforms from which their expression is driven, complementing the type I IFNs solely induced upon mitochondrial signaling [34]. 

The MAVS-specific signaling from distinct organelles was more recently contested by another group [21]. They have reported that the activation of MAVS in either the peroxisomes or mitochondria induces the expression of both type I and type III IFNs, in similar levels. Moreover, they suggest that the absence of peroxisomes does not affect the capacity of cells to mount an effective antiviral response. These contradictory results may be due to the distinct experimental setups, cell lines, and methodologies used, but should certainly be clarified in the near future. Nevertheless, the fact that distinct viruses have developed specific strategies to target and evade the peroxisomal antiviral signaling (discussed in the next section of this review) certainly highlights the significance of this organelle in the context of the cellular antiviral immune response. Furthermore, the specific metabolic and morphological differences between peroxisomes and mitochondria are likely to be responsible for particular differences between these signaling mechanisms, such as distinct interactors of MAVS or adaptations to the different virus life cycles.

### Viral Evasion of the Peroxisome-Dependent Antiviral Response

The important role of peroxisomes as signaling platforms in RLR antiviral immunity is supported by numerous studies that report the specific evasion of peroxisome-dependent signaling by different viruses. It has been demonstrated that the human cytomegalovirus (a virus with a slow replication cycle that has developed highly sophisticated immune evasion strategies [36]) specifically highjacks the transport machinery of the peroxisomal membrane protein in order to transport its own protein, viral mitochondrial-inhibitor of apoptosis (vMIA), to this organelle [37]. At peroxisomes, vMIA interacts with MAVS and inhibits peroxisome-dependent antiviral signaling. vMIA has previously been found to induce mitochondrial fragmentation and, consequently, inhibit mitochondria-dependent signaling [38]. Importantly, although peroxisomes also fragment in the presence of this protein, it was shown that this morphology change is not essential for vMIA’s inhibition of the signaling from this organelle [37].

Different groups have also demonstrated that the hepatitis C virus protein complex NS3-4A localizes at peroxisomes, cleaving MAVS at the organelle’s surface, and impairs the production of ISGs, as it had previously been shown for mitochondria and MAMs [21,28,30,39]. 

Dengue and West Nile viruses were also shown to impair peroxisome biogenesis and dampen the early innate immune signaling from peroxisomes, through PEX19 sequestration by their capsid proteins [40]. 

Herpes simplex virus 1 was also observed to evade the peroxisomal MAVS-dependent signaling through the viral protein VP16, via a mechanism that has not yet been unveiled [41]. 

Additionally, N^pro^ from pestiviruses was reported to localize at peroxisomes, alongside with IRF3 and ubiquitin, inducing IRF3 degradation and inhibiting the downstream antiviral signaling [42]. 

Table 1 summarizes the above-mentioned strategies of evasion of the peroxisome-dependent antiviral response by different viruses.

Some other viruses have been described to interfere with the peroxisome-dependent antiviral signaling, although specific mechanisms of evasion have not yet been disclosed. Upon human immunodeficiency virus (HIV) infection, secondary structured HIV-derived RNA was detected at peroxisomes and induced IRF1 and IRF3 activation, as well as NF-кB, with apparently low expression of type I and III IFNs however [43]. Additionally, it was described that HIV infection upregulates miRNAs that target essential genes required for peroxisomal biogenesis. While MAVS was one of the targets of these miRNAs, no further studies to understand if HIV modulated the peroxisome-dependent antiviral signaling were performed. Curiously, the transfection of miRNAs that target *PEX* genes led to an increase of the mRNA levels of several innate immunity genes [44]. Hepatitis B virus was also described to induce NF-кB due to the targeting of its protein HBx to peroxisomes [45]. 

**Figure 2:**
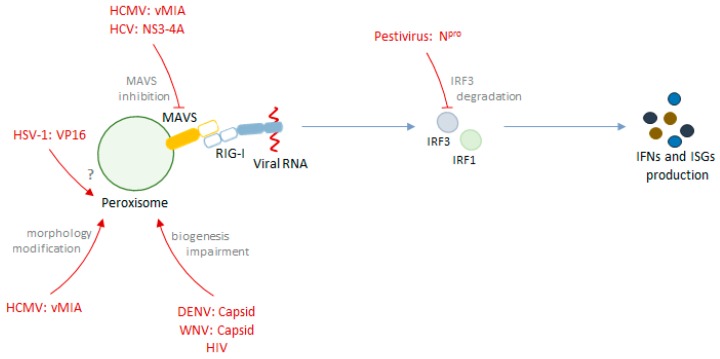
Schematic representation of the interplay between different viruses and the peroxisome-dependent antiviral signaling.

Figure 2 summarizes the above-mentioned mechanisms of interplay between different viruses and the peroxisome-dependent antiviral response.

## 3. Peroxisomes and the Antimicrobial Immune Response: Beyond Viral Restriction

While the role of peroxisomes in innate immunity gained more visibility with the discovery of the localization of MAVS at this organelle, peroxisomes had already been implicated in other innate immunity processes. In 1979, Eguchi et al. proposed that, during phagocytosis in rat peritoneal macrophages, peroxisomes relocated to regions juxtaposed to phagosomes in order to discharge catalase [46]. Catalase, a peroxisomal enzyme with bactericidal activity in the presence of hydrogen peroxide, had been previously identified in the phagocytic vesicle fraction of lysed alveolar macrophages [47]. Moreover, it has been shown that phagocytosis induction increases peroxisome numbers [46].

Later, a study with *Drosophila* and animal cells with impaired PEX5 and PEX7 revealed that peroxisomes are essential for the eradication of microbial infections. The impaired cells were incapable to react to microbial pathogens, presenting defects in immune signaling and reduced viability [48]. Both *Drosophila* and murine macrophages have shown compromised phagocytosis due to defects on actin organization, as well as lysosome formation and/or maturation, which was shown to be associated with the accumulation of ROS andreactive nitrogen species (RNS). Moreover, treatment of macrophages with peroxisome-derived lipids enhanced the capacity of macrophages to engulf bacteria [48]. Similarly, Facciotti et al. demonstrated that the same type of lipids is essential for the maturation of invariant natural killer T (NKT) cells in the thymus [49].

More recently, Di Cara et al., using *Drosophila* as an animal model, revealed that peroxisomes are essential platforms in the maintenance of enteric health and the functionality of the gut–microbe interface, efficiently coordinating different mechanisms such as stress, metabolic and immunity signaling pathways. Moreover, impaired metabolic signaling led to an increase of autophagy-induced epithelial cell death, and the reduced immune response led to a decrease in the reactivity to a subsequent immune challenge and early death [50].

Additionally, Odendall et al. have shown that, in the context of infection with *Listeria monocytogenes* (a bacteria that is signaled by the RIG-I/MAVS pathway and induces mitochondrial disruption [51,52]), peroxisomal MAVS has a dominant role in the coordination of an IFN response, since the expression of peroxisomal MAVS strongly potentiates the production of type I and type III IFNs in Jeg3 trophoblasts [34].

These results provide clear evidence that peroxisomes are not only essential for antiviral immunity but also for the elimination of other microbes such as bacteria.

## 4. Peroxisomes and Inflammation

Inflammation comprises different mechanisms that allow the host to respond to infections and tissue damage, promoting pathogen destruction and wound healing. Upon infection, receptors of the innate immune system activate the production of a variety of proinflammatory mediators. These components, in turn, elicit a local inflammatory exudate, which consists of plasma proteins and leukocytes. At these sites, direct contact with pathogens or cytokines activates neutrophils, culminating with the release of toxic content of neutrophil granules, such as ROS and RNS, which have an unspecific targeting towards pathogens and the host (reviewed in [53]).

Peroxisomes’ role on the elimination of ROS and RNS species establishes a connection between this organelle and inflammation, since catalase and peroxiredoxins, besides neutralizing ROS generated during β-oxidation of lipids, are also essential for maintaining cellular redox homeostasis [17,54,55]. It was shown that tumor necrosis factor-alpha (TNF-α), a proinflammatory cytokine rapidly released upon infection or trauma [56], suppresses peroxisomal β-oxidation in rat hepatocytes and downregulates the expression of mRNAs encoding for peroxisomal proteins such as catalase and acyl-CoA oxidase [57,58].

Peroxisomes were also shown to metabolize leukotrienes and prostaglandins, important modulators of inflammation [59,60,61]. The inactivation of these proinflammatory lipids through β-oxidation produces metabolites that can act as resolution mediators of inflammation [17,61]. Supporting this, Vijayan et al. have also shown that the induction of peroxisomal proliferation in macrophages dampens lipopolysaccharide (LPS)-induced proinflammatory cytokines, while the disruption of their function has the opposite effect, leading to a hyper-induction of these cytokines. Furthermore, they suggest that the up-regulation of peroxisomal proliferation may serve as an auto-regulatory mechanism in macrophages, which renders protection against uncontrolled activation. With this, peroxisomes may act as late-phase inflammation suppressors at the post-translational level, to self-regulate inflammatory macrophages [62].

The loss of peroxisomal functions has also been associated with an intensification of the inflammatory response that can be explained by an accumulation of arachidonic acid metabolites, observed in different models of pathology [63]. For example, it was suggested that loss of peroxisomal β-oxidation from non-neural cells (e.g., microglia and/or infiltrating monocytes) worsens the inflammatory state of the brain [64,65].

## 5. Conclusions and Future Perspectives on the Role of Peroxisomes in Innate Immunity

Peroxisomes are no longer considered mere metabolic organelles, and they are now widely recognized as signaling hubs and protective organelles with significant physiological functions and impacts on many important human diseases. Their emergence as regulators of the innate immune response against viral infections has raised the interest in this organelle, and a growing body of evidence demonstrates that different viruses have developed specific mechanisms to counteract the peroxisome-dependent antiviral response. However, the specificities of this organelle’s dynamics that influence these immune responses needs to be further clarified. Moreover, it is also unknown whether inter-organelle interactions are involved in the establishment of antiviral signaling pathways. Finally, it remains unclear whether other antiviral signaling pathways (besides the RIG-I/MAVS network) may also operate from peroxisomes. Further studies may reveal peroxisome- dependent host mechanisms that can be exploited not only to the discovery of specific viral combat strategies but also to the potential development of broad-spectrum antiviral therapeutics.

**Figure 3:**
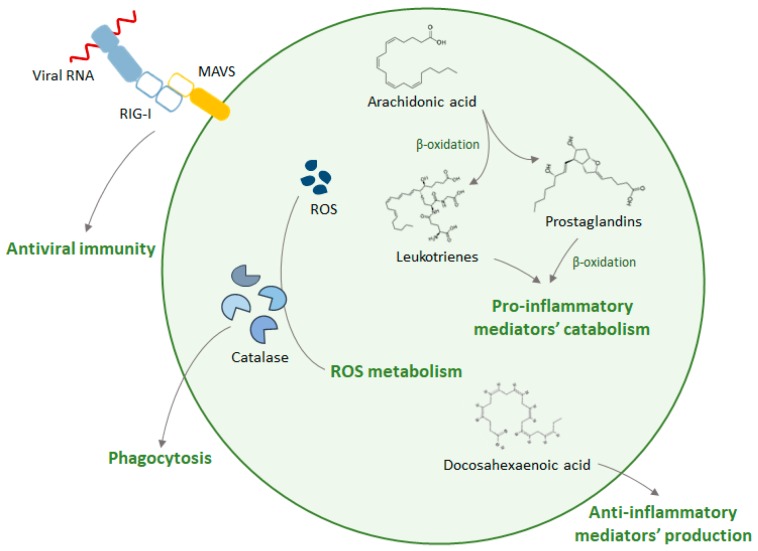
Schematic representation of the peroxisomal functions within innate immunity. Peroxisomes play an important role in antiviral defense through the RIG-I/MAVS-dependent signaling. Additionally, peroxisomes function as anti-inflammatory platforms as they metabolize and produce, respectively, proinflammatory and anti-inflammatory mediators. Moreover, peroxisomes discharge catalase into phagosomes, which is essential for reactive oxygen species (ROS) metabolism during inflammation.

As discussed above and summarized in Figure 3, the involvement of peroxisomes in innate immune mechanisms goes beyond the antiviral response, as they have also been shown to coordinate antimicrobial defenses against bacteria and act as anti-inflammatory platforms.

A further understanding of the mechanisms involved in the role of peroxisomes in innate immunity and inflammation may not only disclose new targets for antiviral and/or antibacterial therapy, but it may also prove beneficial for therapeutic interventions in chronic inflammatory disorders.

## Figures and Tables

**Table 1:** Viral evasion strategies that target peroxisome-dependent antiviral signaling.

Virus	Viral Protein	Mechanism	Cell Type	Ref.
Human cytomegalovirus	viral mitochondrial-inhibitor of apoptosis (vMIA)	Interaction with MAVS	MEFs	[37]
Hepatitis C virus	NS3-4A	Cleavage of MAVS	MEFs, Huh7, A549, HEK293T	[21,39]
Herpes simplex virus 1	VP16	Unknown	HEK293, MEFs, HEK293T	[41]
Dengue virus and West Nile virus	Capsid	Peroxisome biogenesis impairment	A549, HEK293T	[40]
Pestiviruses	N^pro^	Induction of IRF3 degradation	MEFs	[42]
